# Temperature-Induced Viral Resistance in *Emiliania huxleyi* (Prymnesiophyceae)

**DOI:** 10.1371/journal.pone.0112134

**Published:** 2014-11-18

**Authors:** B. Jacob Kendrick, Giacomo R. DiTullio, Tyler J. Cyronak, James M. Fulton, Benjamin A. S. Van Mooy, Kay D. Bidle

**Affiliations:** 1 Grice Marine Laboratory, College of Charleston, Charleston, SC, United States of America; 2 Department of Marine Chemistry and Geochemistry, Woods Hole Oceanographic Institution, Woods Hole, MA, United States of America; 3 Environmental Biophysics and Molecular Ecology Group, Institute of Marine and Coastal Sciences, Rutgers University, New Brunswick, NJ, United States of America; German Primate Center, Germany

## Abstract

Annual *Emiliania huxleyi* blooms (along with other coccolithophorid species) play important roles in the global carbon and sulfur cycles. *E. huxleyi* blooms are routinely terminated by large, host-specific dsDNA viruses, (*Emiliania huxleyi* Viruses; EhVs), making these host-virus interactions a driving force behind their potential impact on global biogeochemical cycles. Given projected increases in sea surface temperature due to climate change, it is imperative to understand the effects of temperature on *E. huxleyi*’s susceptibility to viral infection and its production of climatically active dimethylated sulfur species (DSS). Here we demonstrate that a 3°C increase in temperature induces EhV-resistant phenotypes in three *E. huxleyi* strains and that successful virus infection impacts DSS pool sizes. We also examined cellular polar lipids, given their documented roles in regulating host-virus interactions in this system, and propose that alterations to membrane-bound surface receptors are responsible for the observed temperature-induced resistance. Our findings have potential implications for global biogeochemical cycles in a warming climate and for deciphering the particular mechanism(s) by which some *E. huxleyi* strains exhibit viral resistance.

## Introduction

*Emiliania huxleyi* (Lohman) Hay & Mohler is a cosmopolitan, coccolithophorid haptophyte known to form dense annual blooms, with reported concentrations ranging from 10^6^ to 10^8^ cells per mL [1], [2]. These blooms are large enough to impact both the global carbon and sulfur cycles. *E. huxleyi* largely impacts the sulfur cycle through the production of dimethylated sulfur species (DSS) like dimethylsulfoniopropionate (DMSP) and dimethylsulfide (DMS), because of this *E. huxleyi* is one of the most well studied and ecologically relevant species of marine phytoplankton.

Algal DMSP is converted into DMS and acrylate primarily via the activity of the enzyme DMSP lyase, which is produced by both phytoplankton and bacteria [3], [4]. DMS is detectable in the surface water column, and is a highly volatile compound that easily diffuses into the atmosphere. Through mechanisms such as photo-oxidation, DMS is converted into sulfate aerosols which represent the largest biogenic source of cloud condensation nuclei (CCN). CCN production increases the earth’s cloud albedo, thereby significantly impacting the earth’s radiation budget [5], [6].

In recent years the ecological importance of marine algal viruses in controlling phytoplankton community structure and succession has become a topic of great interest among phycologists, oceanographers, and biogeochemists [7]–[15]. In fact it is now generally accepted that *E. huxleyi* blooms are often terminated by a host-specific virus, *Emiliania huxleyi* Virus (EhV) [9], [13], [16]. This phenomenon has also been seen in the harmful bloom-forming Raphidophyte, *Heterosigma akashiwo* [17].

Several possible resistance mechanisms have been proposed based on physiological differences between resistant host strains and their sensitive counterparts. For example, some *E. huxleyi* strains may switch to a haploid life stage that is physiologically impervious to virus infection, through an as yet unidentified mechanism [18], [19]. Likewise, observations of successful virus entry and subsequent recovery of a resistant strain (Center for the Culture of Marine Phytoplankton, CCMP 373) implicate a subcellular ‘molecular armor’ revolving around regulation of the programmed cell death (PCD) machinery, which EhVs activate and recruit for successful infection [20]. Elevated DSS production in resistant strains has also been proposed as a mechanism for viral resistance [21], presumably through its role as a subcellular antioxidant. Indeed, EhV infection is known to cause late lytic-phase oxidative stress in *E. huxleyi* [22], [23] and cellular DSS compounds such as DMS, DMSP, dimethylsulfoxide (DMSO), and their metabolites have established antioxidant roles in algal cells [24]. Steinke et al. [25] characterized resistant *E. huxleyi* strains (CCMP 373 and 379) as having high DMSP lyase activity, which could lead to elevated intracellular antioxidant capacity due to the free radical scavenging ability of DMS [24]. If the oxidative stress induced by infection makes the host cell more susceptible to viral replication then an elevated antioxidant capacity due to higher DMSP-lyase activity could mitigate oxidative stress to levels beneath the threshold required for host lysis.

The antioxidant function of DSS can be summarized by the DMSP antioxidant cascade described by Sunda et al. [24]. Under periods of oxidative stress, DMSP can be oxidized or enzymatically cleaved into DMS and acrylic acid, both of which have been shown to decrease infectious titers of EhV [21]. DMS can be oxidized by hydroxyl radicals to DMSO and finally to methanesulfinic acid [24]. Many species of phytoplankton, including *E. huxleyi*, can reduce DMSO back to DMS allowing the process to repeat [26]. Additionally, several steps in the pathway result in a higher affinity for hydroxyl radicals than DMSP [24] making this a very effective pathway for relieving oxidative stress. The DMSP antiviral hypothesis put forth by Evans et al. [21] was later framed as a mechanism by which virally lysed cells can lead to a decrease in the infectivity of subsequent viral progeny, allowing a subset of the population to temporarily resist viral mortality and delay bloom termination [23]. This may have important climatic impacts as *E. huxleyi’s* contribution to atmospheric sulfur may be dependent on delayed bloom termination.

Another potential mechanism for viral resistance involves the lipid composition of *E. huxleyi* cells. EhVs employ a sophisticated, co-evolutionary “arms race” to manipulate host lipid metabolism, most notably glycosphingolipid (GSL) production, and critically regulate infection and cell fate via activation of PCD [20], [23], [27], [28]. EhVs possess a lipid envelope in addition to a protein capsid, and they contain a suite of glycosphingolipid biosynthetic genes within their genome [8], [29]–[31]. Furthermore, recent work has shown that the viral lipid envelope is comprised almost entirely of GSLs that appear to be acquired by budding from lipid rafts on the host cell plasma membrane [28], [32]–[34]. Resistant strains appear to lack a specific GSL with a sialic acid headgroup (sGSL) that are enriched in purified lipid raft fractions [33] and may play a role in virus attachment or release [32]. In addition, while these host-derived GSLs (hGSLs) can serve to influence cellular response to EhV infection, simultaneous synthesis of virus-derived GSL (vGSL) molecules ultimately regulates the synthesis of viral progeny [27], [28], [33]. The subcellular processes that regulate the production of vGSLs ultimately control the induction of reactive oxygen species (ROS), PCD, and dynamics of viral infection [27], [28].

Here, we examine the impact of temperature on *E. huxleyi*-EhV interactions. Although the Intergovernmental Panel on Climate Change (IPCC) predicts a 1.5–3°C increase in sea surface temperature by the year 2100 [35], its impact on the biology and ecology of this host-virus system remains unexplored. Temperature has a well-known impact on lipid membrane composition and fluidity [36] and, given the importance of lipids to the aforementioned *E. huxleyi*-EhV ‘arms race’, it likely impacts infectivity. There is precedence for a temperature influence on virus infectivity. For example, the algicidal activity of *H. akashiwo* Virus (HaV) varies with only a few degrees change in temperature [17]. We specifically show that host resistance to EhV86 is induced by only a 3°C elevation in incubation temperature, and that successful infection leads to changes in DSS and polar membrane lipid composition and dynamics. Our findings shed light on the interactive effects of elevated temperature and viral infection on a globally important phytoplankton species and provide corresponding context into its impact on the biogeochemical cycling of carbon and sulfur in *E. huxleyi* bloom areas.

## Materials and Methods

### Experimental Cultures

Axenic cultures of *Emiliania huxleyi* CCMP 374, isolated from the Gulf of Maine and obtained from the National Center for Marine Algae and Microbiota (formerly CCMP), were maintained at 18°C in L1 medium (-Si) on a 12/12 light/dark cycle and irradiance of 57 µE m^−2^s^−1^. Growth media was mixed and sterilely transferred to 1L polycarbonate bottles, six of which were kept at 18°C and six at 21°C. Since the IPCC predicts a 1.5–3°C increase in global mean temperature by the year 2100 [35], a 3°C increase was chosen for these experiments. Bottles were inoculated to 2.5*10^4^ cells ml^−1^ and cell density was monitored until cultures reached roughly 4*10^5^ cells ml^−1^. Cultures were then subjected to infection with EhV86 at an average multiplicity of infection (MOI) of 5 (day 0). Uninfected cultures were grown at each temperature to serve as controls, and each treatment or control was replicated three times for a total of 12 bottles.

### Sampling Protocol, Cell and Virus Abundance, and Physiological Status of Host

Cultures were sampled daily in a Class 100 laminar-flow hood under sterile conditions by transferring 150 mL into amber polyethylene bottles from which subsamples were taken and processed as described. One mL was diluted in filtered sea water and analyzed on a Coulter Counter (Beckman Coulter Multisizer III) for culture density and cell biovolume. Viral abundance was measured via flow cytometry following the SYBR Green staining protocol [37]. Briefly, two mL were fixed with 40 µL of 25% glutaraldehyde at 4°C in the dark for 30 min. Samples were then flash frozen in liquid nitrogen and stored at −80°C until analysis on a Beckman Coulter MoFlo Astrios flow cytometer (FCM). Viral particles were enumerated using side scatter and SYBR green fluorescence (513±20 nm). The cellular induction of ROS was also measured via FCM by treating one mL of culture with 5-(and-6)-chloromethyl-2′,7′-dichlorodihydrofluorescein diacetate (CM-H_2_DCFDA) to a final concentration of 5 µM and incubated for 60 min in the dark. Cellular ROS were visualized and quantified based on the mean fluorescence at ∼522 nm following the protocol of Evans et al. and Vardi et al. [22], [23]. To assess the photochemical quantum yield of photosystem II (PSII; F_v_/F_m_), five mL were dark adapted for 15 min, and F_v_/F_m_ was measured using a Walz Phyto-C PHYTO-PAM fluorometer.

### Dimethylated Sulfur Species

To measure total DMSP (DMSP_t_) and total DMSO (DMSO_t_) concentrations, five mL were taken from each sample, acidified with 25 µL of 50% H_2_SO_4_ to prevent conversion of DMSP into DMS, and stored at 4°C until analysis. For dissolved pools of DMS, DMSP, and DMSO, approximately 25 mL were gravity filtered through a Whatman GF/F filter, collected in 25 mL plastic scintillation vials with no headspace, and stored on ice. DMS was measured immediately (typically within 6 hours of sampling). All samples were measured using a Hewlett Packard 5890 Series II gas chromatograph equipped with flame photometric detection. Samples were sparged with helium (125 mL/min) for at least 25 min and collected on glass fibers in a Teflon loop submerged in liquid nitrogen. Once collected, the sample was heated to ∼80°C and injected into the gas chromatograph [38].

Total and dissolved DMSO samples were base hydrolyzed in 2N NaOH to convert DMSP to DMS (volumes varied with cell density) and sparged with ultra-high purity (UHP) nitrogen for 1 hour to remove the DMS. Samples were then transferred to a purge and trap manifold, sparged with UHP helium for an additional five minutes, and analyzed by gas chromatography to insure all DMSP was removed. If residual DMSP remained, samples were sparged with helium again at 5 minute intervals and re-analyzed until DMSP was undetectable. A cobalt-doped sodium borohydride tablet (Sigma-Aldrich) was then dropped into the sparging chamber and the collection loop was opened without starting the sparging helium to prevent over-pressurizing the chamber [39]. After five minutes, the sparging helium was turned on and the sample run as described above. Particulate pools of DMSP (DMSP_p_) and DMSO (DMSO_p_) were calculated by subtracting dissolved values from total values.

### Intact Polar Lipids

Samples for intact polar lipid (IPL) analysis, including polar glycerolipids and GSLs, were taken by filtering 15 mL through a precombusted GF/F filter, flash freezing the filters, and storing them at −80°C prior to analysis following the high performance liquid chromatography/mass spectrometry (HPLC/MS) protocol of Van Mooy and Fredricks [40]. IPLs were extracted using a mixture of polar and non-polar solvents [41], [42] and were analyzed on a Hewlett Packard 1100 HPLC and LCQ Deca XP ion-trap mass spectrometer. Polar glycerolipids were quantified using molecular ion peak areas as described by Van Mooy and Fredricks [40] and GSLs were quantified by comparison with a response factor for glucocerebrosides (soy, Avanti Polar Lipids) as described by Fulton et al. [33].

### Additional Strains and Temperatures

A series of experiments was performed in which six *E. huxleyi* strains (CCMP 373, 374, 379, 392, 1516, and DWN 61/87/10) were infected with EhV 86 at four temperatures (12°, 15°, 18°, and 21°C) in order to assess whether the temperature effect was shared in a variety of strains. *E. huxleyi* cultures were grown at the experimental temperature for at least one week prior to beginning the experiment. Six replicate 50 mL vials of L1 medium (-Si) were prepared for each strain and allowed to reach experimental temperature overnight. Vials were inoculated to an initial cell concentration of ∼10^5^ cells mL^−1^. Cells were allowed to grow and infected at an average MOI of ∼5 once a concentration of 3*10^5^ to 5*10^5^ cells mL^−1^ was reached (day 0). EhVs used for infection had previously been propagated at 18°C. Two mL were sampled daily on days 1 through 4 and again on day 7 when successfully infected cultures had totally cleared. Samples were preserved with glutaraldehyde and stored at −80°C according to Brussaard et al. [37]. Cell and virus concentrations were measured via FCM as described above. All strains were tested at all 4 temperatures except CCMP 1516 and 373. *E. huxleyi* 1516 was only tested at 18°C and 21°C, and CCMP 373 was not tested at 12°C as it would not grow at this temperature.

### Virus incubations, Absorption, and Burst sizes

To test whether elevated temperature affected only the host’s susceptibility to infection, rather than the direct infectivity of the virus itself, EhV86 isolates were incubated for 24 hours at 18°C and 21°C in the dark and used to infect *E. huxleyi* 374 growing at 18°C. Host cell and virus abundance were measured on day 0 (immediately post infection), as well as 3 and 7 days later to test the infectivity of the isolate. Viral abundance was measured by FCM as described and cell abundance was also measured by FCM with cells discriminated based upon side scatter and chlorophyll fluorescence characteristics (∼710±22.5 nm). A separate experiment was performed to assess the rate of viral absorption into the host. Four replicate cultures of *E. huxleyi* 374 were infected at each temperature (18° and 21°C). EhV abundance was measured via FCM (as above) immediately and at 1, 2, 3, 4, and 24 hours post infection.

Burst size, or the number of viral progeny produced per host cell lysed, was calculated from the increase in viral abundance over the corresponding decreases in host abundance during a 24 hour period using the following equation:
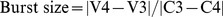



Where V4 and V3 represent viral abundance on days 4 and 3, respectively; C3 and C4 represent cell abundances on day 3 and 4, respectively. All burst sizes were calculated between days 3 and 4 except in strain 1516 which was calculated between days 2 and 3. Days selected represented the latest period in the infection cycle for which measurements were no more than 24 hours apart. Burst size was also calculated for virus incubation data between days 3 and 7 as data were not collected on a 24 hour scale.

### Statistical Analysis

Most data were graphed as a function of time with data points representing the mean of experimental replicates and error bars representing one standard deviation. Data were initially analyzed via a two-way repeated measures (RM) ANOVA using Sigmaplot. When assumptions of normality and/or equal variance were not met (as was frequently the case), a post hoc Holm-Sidak t-test was also used. Lipid data were normalized to cell abundance (fmol cell^−1^) prior to graphing and statistical analysis. Burst size data were compared using a one-way ANOVA (Sigmaplot ver. 11.2, SPSS Inc., Chicago, IL).

## Results

### Temperature, Cell Growth, and Viral Propagation

**Figure 1:**
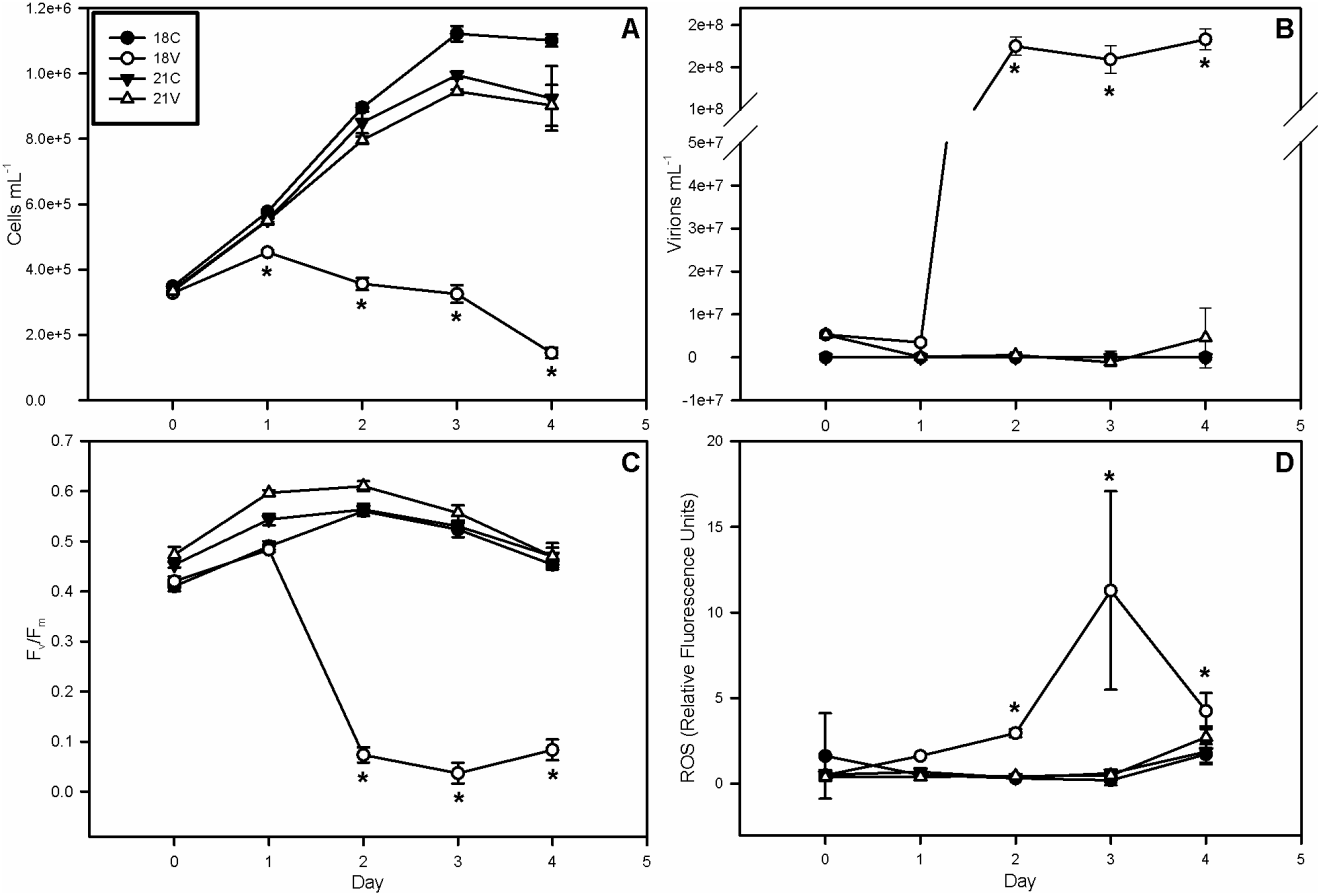
Cell Abundances, Virus Abundances, and Host Health. (A) Mean cell abundance, (B) mean virus abundance, (C) photosynthetic efficiency of photosystem II, (D) and reactive oxygen species (ROS). Host cell abundance and photosynthetic efficiency decrease in the 18V treatment ∼24 h post infection, concomitant with a dramatic increase in viral abundance and accumulation of ROS. All are indicative of active viral infection. The decrease in ROS on day 4 could be due to free radical scavenging by DMSP and its metabolites or a drop-off in late phase viral replication. Error bars represent one standard deviation, and asterisks represent statistical significance according to a Holm-Sidak t-test (p<0.05).

All experimental cultures had an average initial (day 0) concentration of approximately 3.4*10^5^ cells mL^−1^ (Fig. 1A). Control (C) cultures grown at 18°C (hereafter referred to as 18C) reached ∼1.1×10^6^ cells mL^−1^ by day 3, while 21°C control cultures (21C) reached ∼1*10^6^ cells mL^−1^. Virus-infected (V) cultures grown at 18°C (18V) showed a depressed growth rate within 24 hours of infection and were 20% less dense than all other cultures by day 1. Cell densities in 18V decreased rapidly thereafter to a final concentration of 1.5*10^5^ cells mL^−1^, a net decline of more than 50% over the course of the experiment and ∼85% lower than all other treatments on day 4. A 2-way RM ANOVA revealed significant differences between 18V and all other treatments on days 1 through 4 (p<0.05). Viral abundances (Fig. 1B) in both 18V and 21V averaged 5.3*10^6^ virions mL^−1^ on day 0, but only showed rapid and substantial increases (by 33-fold) in 18V with concentrations reaching 1.75*10^8^ virions mL^−1^ by day 2. Viral abundance in 21V was not significantly different from 18C and 21C (*i.e.* 0 virions mL^−1^), indicating that successful EhV infection and propagation did not occur at 21°C (Fig. 1B).

### Physiological Status of Host and Oxidative Stress

The photochemical quantum yield of PS II was measured as the ratio of variable fluorescence to maximum fluorescence (F_v_/F_m_) and used as a proxy of host photosynthetic health. F_v_/F_m_ is one of the first physiological parameters to change upon EhV infection of *E. huxleyi* cells [20], [28]. F_v_/F_m_ in 18V fell to <0.1 by day 2 while the other cultures remained at or above an average of 0.5, an 80% decrease in photosynthetic efficiency (Fig. 1C). Hence, viral infection in 18V significantly (p<0.05) compromised photosynthetic competency in PSII. Concurrent with the decrease in photosynthetic efficiency and cell abundance in 18V was a 22-fold increase in cellular ROS staining from 0.5 relative fluorescence units per cell (rfu cell^−1^) to 11 rfu cell^−1^ by day 3 (Fig. 1D). ROS in 18V then fell by 48% to 4.3 rfu cell^−1^. All other treatments remained at ∼0.5 rfu cell^−1^ throughout the experiment.

### Transmission Electron Microscopy

**Figure 2:**
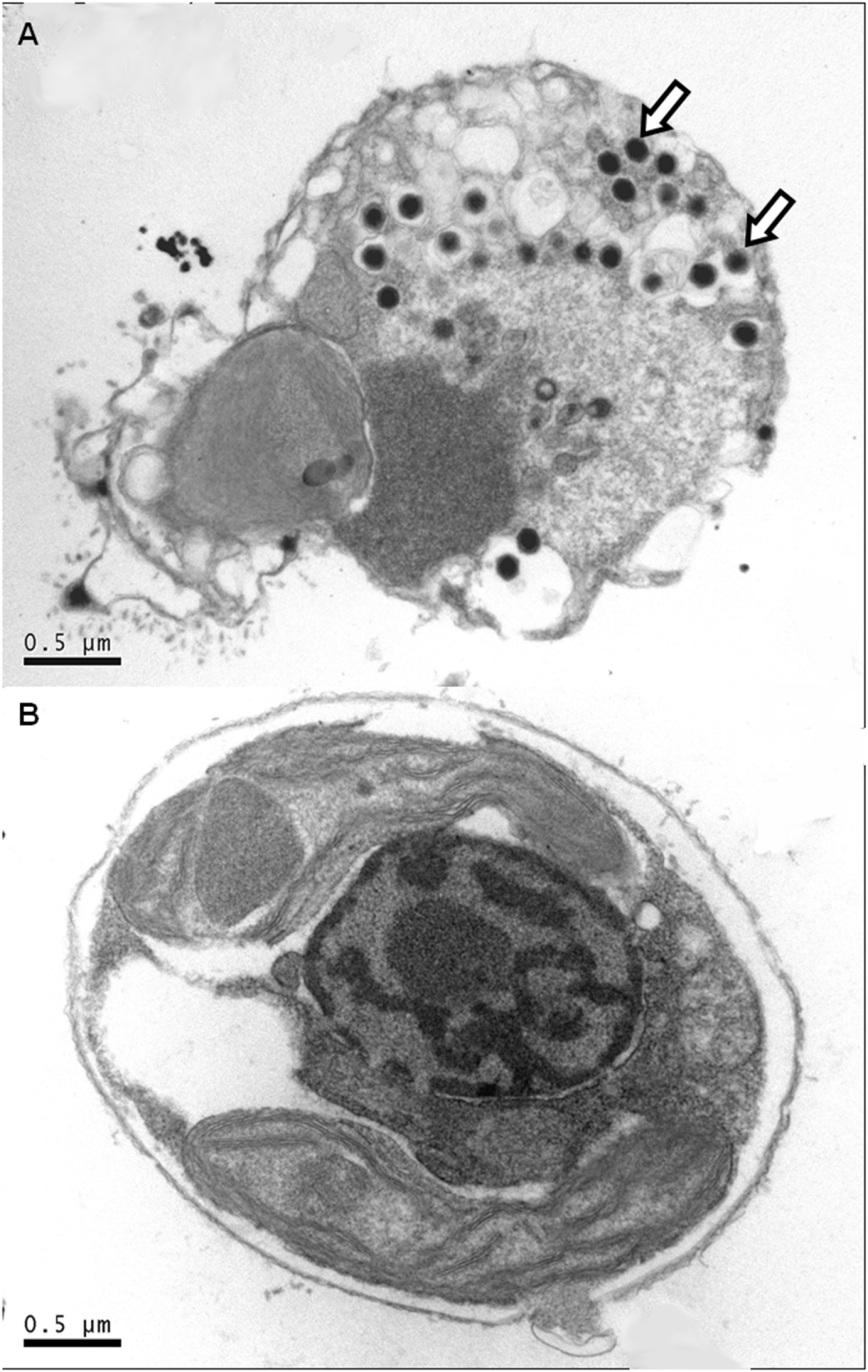
Visual Confirmation of Infection. TEM images of E. huxleyi cells from treatments (A) 18V and (B) 21V taken on day 2 of sampling. Arrows indicate viral particles. The absence of viral particles in B demonstrates that the virus could not cross the host plasma membrane at 21°C.

Electron micrographs were made of preserved cells taken from all treatments on day 2 of the experiment (Fig. 2). Electron dense, intracellular, icosahedral virus particles (indicated by arrows) were present only in 18V (Fig. 2A). These results are consistent with previously published EhV morphology [8] and are indicative of a successful EhV infection in this treatment. Notably, no EhV particles were observed to be incorporated or attached to host membranes in the 18C, 21C (See Fig. S1), and 21V samples (Fig. 2B), suggesting that resistance likely involved a compositional change in surface properties and/or receptors.

### Dimethylated Sulfur Species

**Figure 3:**
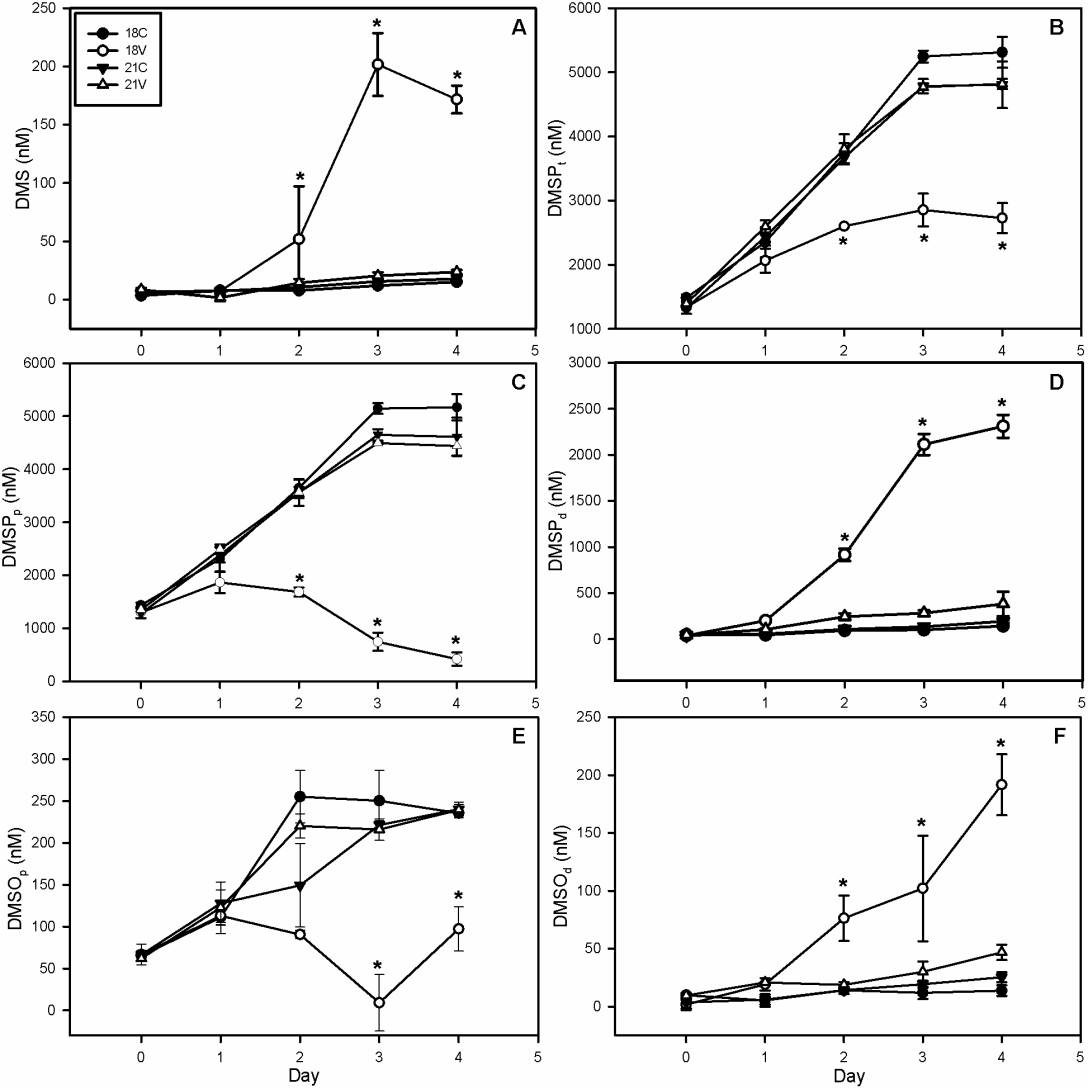
Dimethylated Sulfur Species. (A) Dissolved DMS, (B) total DMSP, (C) particulate DMSP, (D) dissolved DMSP, (E) particulate DMSO, and (F) dissolved DMSO. Dissolved DMS increases by day 3 due to dumping of cellular contents upon host lysis with a decrease in day 4 possibly due to free radical scavenging. Total DMSP and DMSO accumulated more slowly in 18V than other treatments with a decrease in DMSO and DMSP beginning on days 2 and 3, respectively. Particulate pools showed a similar trend, while dissolved pools increased in 18V and remained mostly unchanged in other treatments. If the antioxidant capacity of DSS is responsible for resistance then elevated production of these compounds should be seen in 21C and 21V. Error bars represent one standard deviation, and asterisks represent statistical significance according to a Holm-Sidak t-test (p<0.05). DMS – Dimethylsulfide, DMSP – Dimethylsulfoniopropionate, DMSO – Dimethylsulfoxide. Subscript letters p, d, and t represent particulate, dissolved, and total, respectively.

Dissolved DMS concentrations in 18V increased 29-fold from 7 nM to 202 nM by day 3 (Fig. 3A) indicative of DMS efflux into the culture medium upon host lysis. A subsequent 15% decrease was observed on day 4 to 172 nM, which may partially be due to an increase in DMS oxidation to DMSO (see below). DMS concentrations in the remaining treatments never significantly differed from one another, but increased approximately 3-fold from a mean of 6 nM to 19 nM.

DMSP_t_ showed an attenuated rate of accumulation in 18V compared to all other treatments (Fig. 3B). The maximum concentration of DMSP_t_ in 18V occurred on day 3 reaching 2,853 nM, a ∼2.6-fold increase from day 0. The other 3 treatments exhibited a nearly 5-fold increase in DMSP_t_ to a mean of ∼5,000 nM. DMSP_t_ accumulation in 18V cultures was only 54% that observed in other treatments on day 4. DMSP_p_ accumulated in 18C, 21C, and 21V in a similar manner to DMSP_t_ with an approximately 5-fold increase from ∼1,100 nM to ∼5,000 nM (Fig. 3C). This value calculates to ∼0.77 pg cell^−1^ in 18C, which is similar to the value reported by Keller et al. [43] of 0.75 pg cell^−1^. DMSP_p_ concentration in 18V cultures began to decline by day 2, reaching a minimum of ∼400 nM by day 4, a net ∼64% decrease over the course of the experiment. Conversely, DMSP_d_ increased by 29-fold in 18V reaching 2,309 nM by day 4; all other treatments increased by ∼3-fold on average to 240 nM (Fig. 3D). All three DMSP pools were significantly different in 18V compared to other treatments by day 2 (2-way RM ANOVA, p<0.05).

With the exception of a 30% decrease in 18V between days 2 and 3, all DMSO_t_ concentrations showed a net 3.8-fold increase over the course of the experiment from an initial mean concentration of 71 nM to a final mean concentration of 273 nM (See Fig. S2). The only significant (p<0.05) difference observed in DMSO_t_ was between 18V and all other treatments on day 3. DMSO_p_ in 18C, 21C, and 21V all demonstrated a net ∼5-fold increase from day 0 to day 4 (Fig. 3E). The 18V treatment increased to ∼100 nM for a 2-fold net increase over the course of the experiment, despite a drop to near 0 on day 3 (Fig. 3E). DMSO_d_ increased in 18V to 192 nM, nearly 200-fold, compared to a mean 3.7-fold increase in all other treatments (Fig. 3F). All DSS responses are consistent with the lysis of host cells releasing particulate (*i.e*. intracellular) sulfur into the dissolved phase.

### Viral Absorption

**Figure 4:**
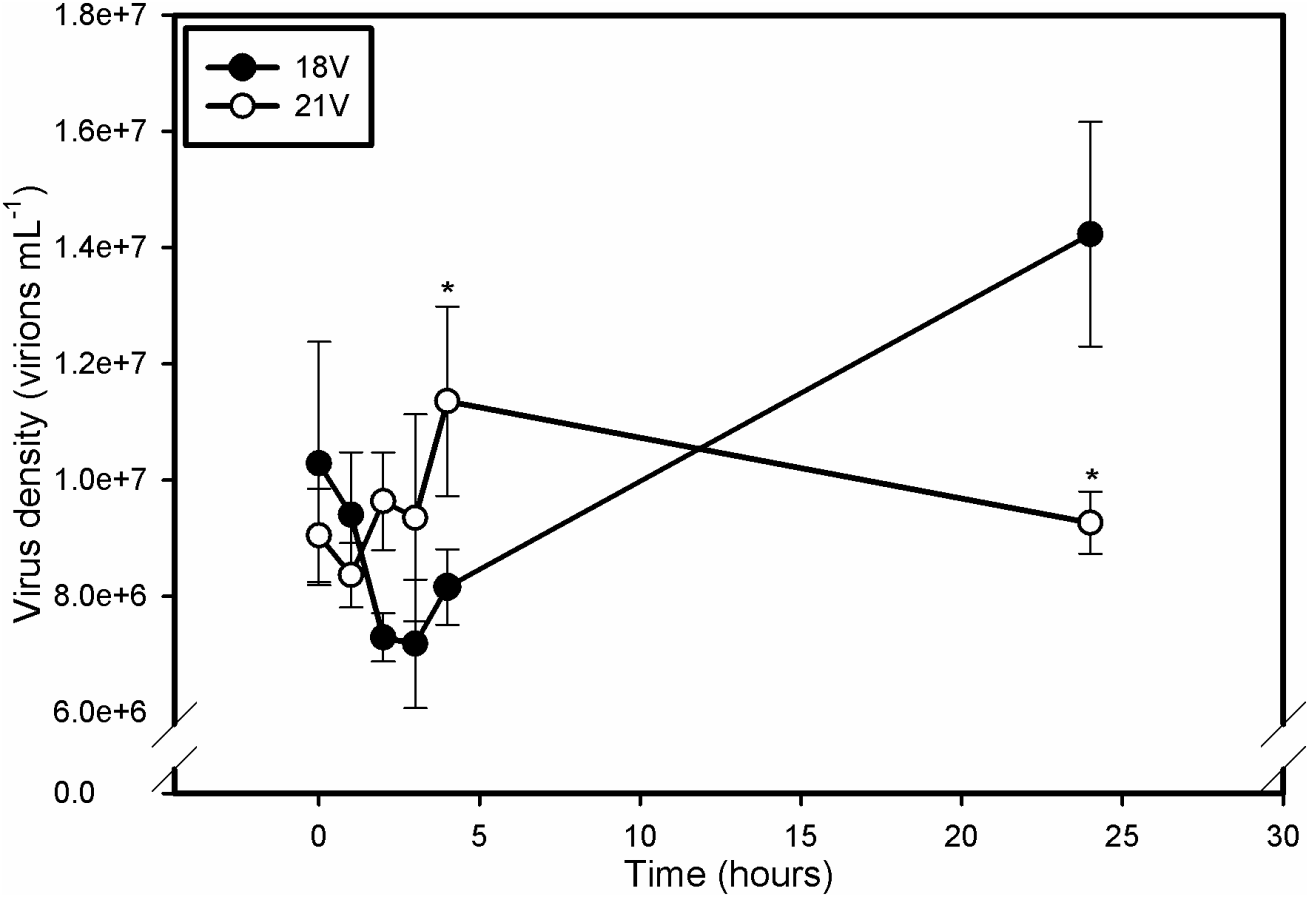
Time course of viral absorption and production. The initial decrease in EhV abundance in 18V represents successful adsorption to and absorption into the host membrane. Emergence of EhV progeny is evident by 4 h. Time-points in 21V are never significantly different from one another, but are different from 18V by hour 4. Resistance of 21V appears to be derived from an inability of the virus to bind to or cross host membranes. Error bars represent one standard deviation, and asterisks represent statistical significance according to a Holm-Sidak t-test (p<0.05). Data were ln transformed for statistical analysis.

EhV absorption showed very different dynamics for 18V and 21V treatments, which support altered virus attachment (Fig. 4). Normally, EhV adsorption to the host cell membrane occurs within 30 minutes, with viral progeny detectable roughly 4 hours post infection [30]. Consequently, successful infection should induce an initial drop in free EhV abundance as virions successfully contact and cross the host plasma membrane, followed by a measurable increase in virus abundance as host cells begin to release viral progeny into the surrounding media. As expected, viral abundance in 18V declined in the first 3 hours, indicating absorption of viral particles into a permissive host followed by increases to 1.4*10^7^ virions mL^−1^ after 24 hours indicative of active infection. At no time was viral abundance significantly different than time 0 in 21V indicating no change in virus concentration. A 2-way RM ANOVA with a post-hoc Holm-Sidak t-test of natural log transformed data revealed that viral abundance was significantly lower in 18V at 4 hours and significantly higher at 24 hours relative to 21V. This result is consistent with successful absorption and infection of 18V. In addition, these results confirm that the higher temperature treatment conferred a first-order resistant phenotype to *E. huxleyi* 374 due to a reduced ability of EhVs to attach to host cell membranes, perhaps due to a change in the content and/or composition of host cell surface receptors (see discussion below).

### Intact Polar Lipids

**Figure 5:**
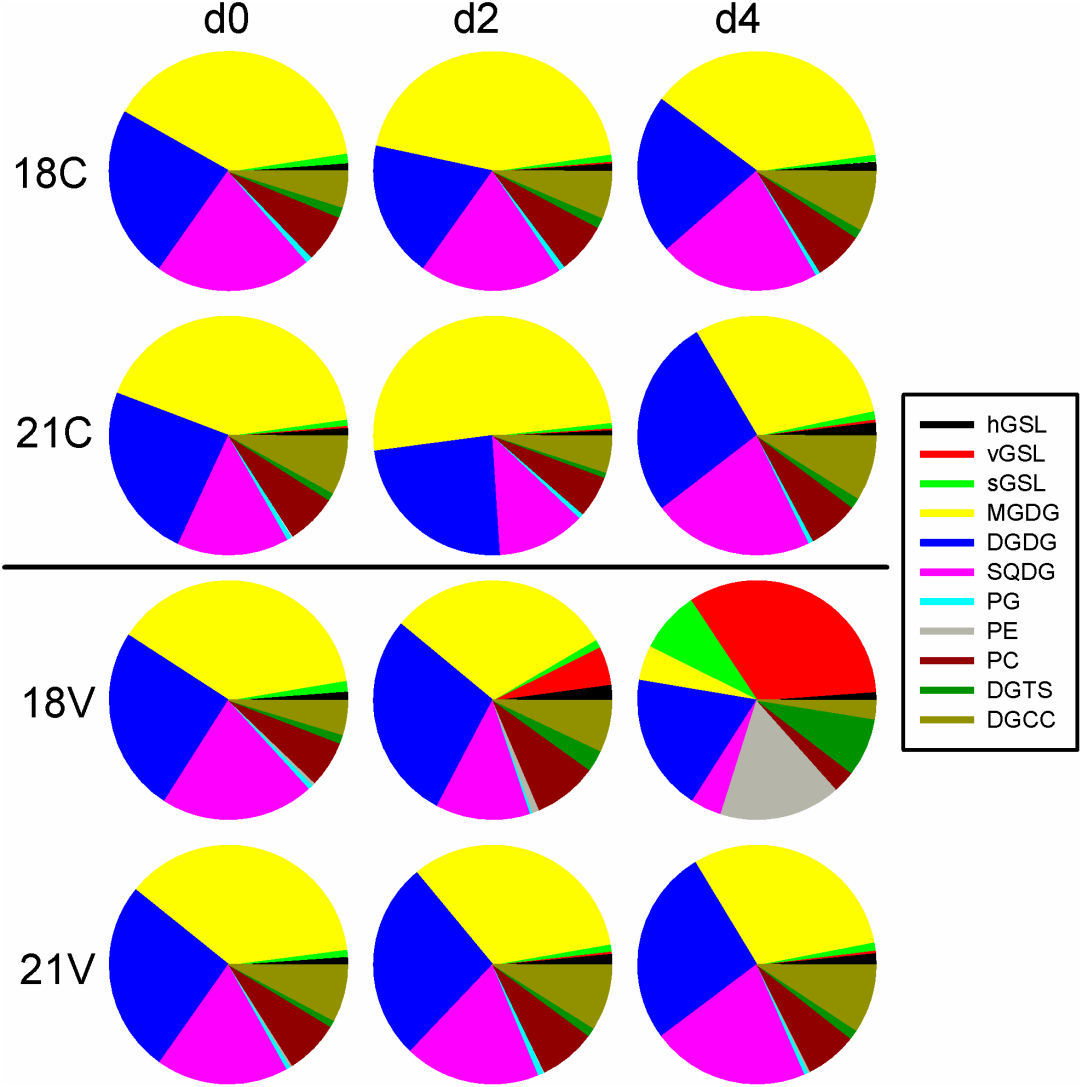
Intact Polar Lipids. Pie charts represent relative concentrations of individual lipids at days 0, 2, and 4 in the respective treatments. Colors are explained in the legend (right). Viral GSLs (red pie slices) accumulated in 18V and not the other treatments again indicating successful infection. Chloroplast lipids such as MGDG (yellow slices), DGDG (blue slices), and SQDG (pink slices) decrease dramatically in 18V as photosynthetic efficiency decreases. 18V also demonstrates a significant decrease in diacylglycerylcarboxy-N-hydroxymethyl-choline (DGCC, dark green slices) which is involved in fatty acid transport into the MGDG synthesis pathway. Host derived GSLs are significantly higher in 21C than in 18C by day 4. GSL – Glycosphingolipid (h, v, and s represent host-derived, virus-derived, and scialic acid, respectively), MGDG – Monogalactosyldiacylglycerol, DGDG – Digalactosyldiacylglycerol, SQDG – Sulfoquinovosyldiacylglycerol, PG – Phosphatidylglycerol, PE – Phosphatidylethanolamine, PC – Phosphatidylcholine, DGTS – Diacylglyceryltrimethylhomoserine, DGCC – Diacylglycerylcarboxyhydroxymethylcholine.

Viral infection has been shown to decrease the IPL content of infected *E. huxleyi* 374 populations by ca. 25% four days post infection [33]. We examined several key lipid groups in 18°C and 21°C cultures, including the three types of polar diacylglycerolipids: phospholipids, glycolipids, and betaine lipids (Fig. 5). Phospholipids included phosphatidylcholine (PC), phosphatidylglycerol (PG), and phosphatidylethanolamine (PE), which contribute to several functional membranes including the outer cell membrane-lipid bilayer [34]. No significant differences were seen in PG abundance; however, a significantly (p<0.05) lower PC concentration per cell was observed in 18V relative to the earlier time points, while PE concentration starting on day 2 was significantly (p<0.05) higher than all other treatments.

Significant (p<0.05) accumulation of all three different GSL classes (sGSLs, hGSLs, and vGSLs) was seen in 18V starting on day 2 (Fig. 5). Similar levels of sGSLs, potential markers of *E. huxleyi* infection susceptibility, were detected in all treatments. While the sGSL concentration increased in 18V throughout the experiment, changes in sGSL content were not observed in 18C, 21C or 21V. Pools of hGSLs in 18V initially increased, becoming significantly higher than other treatments on days 2 and 3 then dropped to nearly zero by day 4 which coincided with an increase in the rate of vGSL accumulation (Fig. 5). However, hGSLs were not significantly different between 18C and 21C at any time point. Significant accumulation of vGSLs was also seen in 18V starting on day 2, while vGSL concentrations did not increase significantly in the other treatments (Fig. 5). Data values for host cell and virus abundance, host physiology, DSS, and IPLs can be found in Table S1.

### Additional Strains and Temperatures

**Figure 6:**
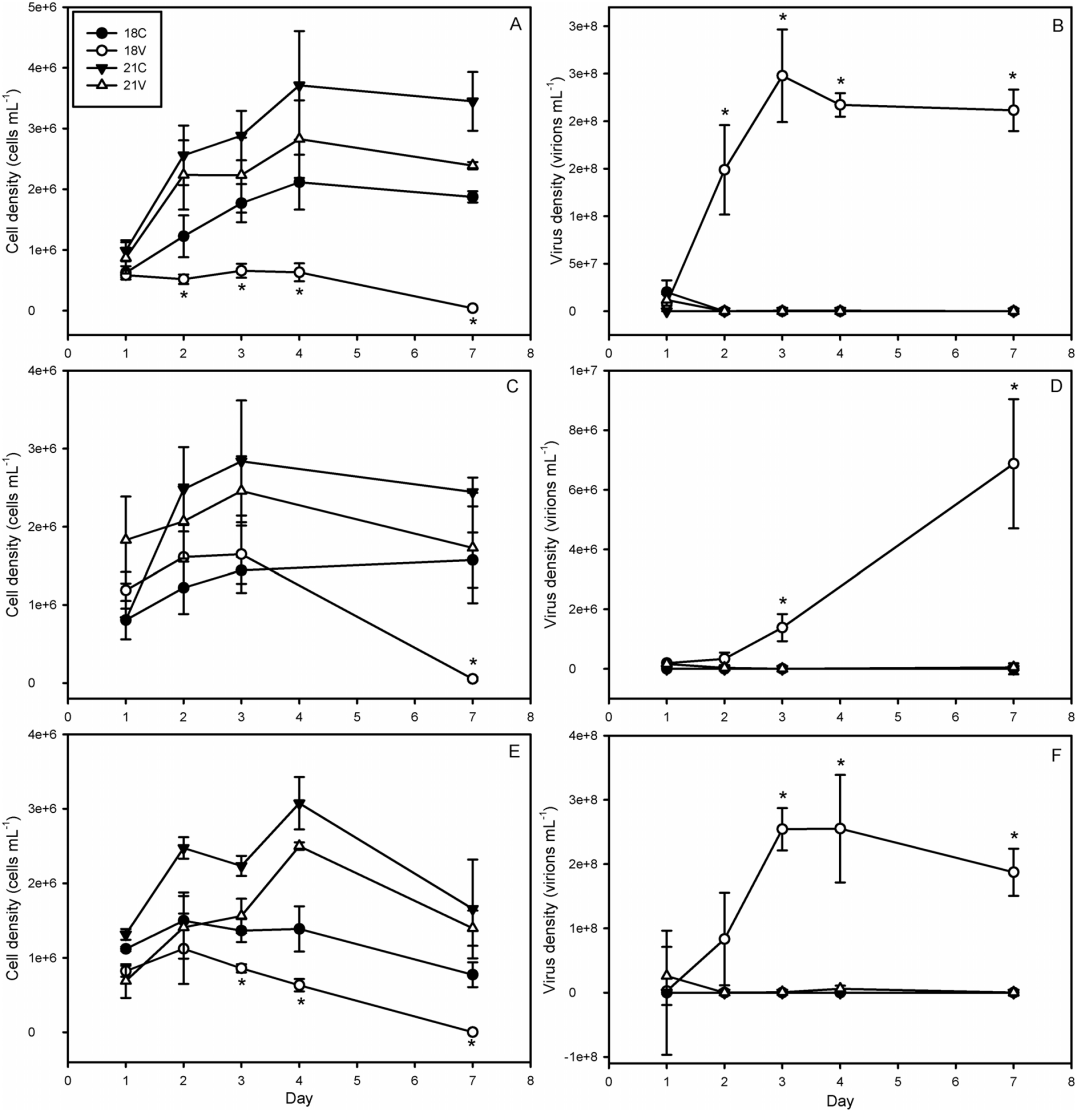
Dynamics of Cell and Virus Abundance for Additional Sensitive Strains. Cell and viral abundance for E. huxleyi strain (panels A and B, respectively) CCMP 374, (C and D, respectively) CCMP 1516, and (E and F, respectively) DWN 61/87/10 at 18° and 21°C. Significant accumulation of viral particles and loss of host cell abundance indicates successful infection. This is only seen in 21V treatments. Error bars represent one standard deviation, and asterisks represent statistical significance according to a Holm-Sidak t-test (p<0.05).

In order to assess whether the observed temperature effect was strain specific, the sensitivity of five other *E. huxleyi* strains was tested at four temperatures. A combination of EhV86-susceptible (374, 61/87/10, and 1516) and EhV86-resistant (373, 379, and 392) strains were tested. These host strains spanned a range of reported DMSP lyase activities, implying differences in efficacy of the putative DMSP antioxidant cascade from strain to strain [22], [24]. These additional experiments confirmed temperature-induced resistance for strain 374 (Fig. 6A, B) and demonstrated nearly identical responses for strains 1516 (Fig. 6C, D) and 61/87/10 (Fig. 6E, F). We classified successful infection as requiring a significant increase in viral abundance concomitant with a significant decrease in cell abundance over the course of the experiment. Host cell abundance in infected cultures of strain 374 decreased by an order of magnitude from day 1 to day 7 at 18°C but not at 21°C (Fig. 6A). Control cell abundances at 21°C were significantly higher than infected cultures (3.4*10^6^ cells mL^−1^ to 2.3*10^6^ cells mL^−1^, respectively) on day 7 (Fig. 6A). Virus abundance showed opposite trends with significant accumulation of viral particles at 18°C but not at 21°C (Fig. 6B).

In strain 1516, 18V cell abundances increased from 1*10^6^ cells mL^−1^ on day 1 to 1.5*10^6^ cells mL^−1^ by day 2 before falling precipitously to ∼0 by day 7 (Fig. 6C). EhV production increased by ∼6 orders of magnitude in 18V during the same time interval (Fig. 6D). Cell abundances at 21°C did not vary significantly from one another but 21C grew 3-fold over the course of the experiment while 21V grew by less than one million cells per mL before decreasing to initial levels by day 7. This attenuated growth in 21V could not be adequately explained by viral mortality alone, as viral abundance was not significantly higher in 21V than 21C by day 7 (Fig. 6D).

Strain 61/87/10 demonstrated similar trends to 374 with significant (p<0.05) decreases in cell abundance and increases in viral concentrations occurring by day 3 at 18°C (Fig. 6E, F). While the 18°C cultures lost cell abundance towards the end of the experiment, they were significantly denser than 18V from days 3 to 7 (Fig. 6E). The significant EhV accumulation (>2*10^8^ virions mL^−1^) classified this result as successful infection (Fig. 6F). The 21C host cell abundances were significantly higher than those of infected cultures until day 7 when both cultures decreased in abundance by nearly 50%. Final cell abundance was at least 5*10^5^ cells mL^−1^ higher by day 7 indicating net growth. Importantly, the final decrease cannot be attributed to viral mortality, as EhV abundance was never significantly higher than 0 (*i.e.* control values). Strains 373, 379, and 392 were resistant at all temperatures tested (See Fig. S3). Cell and virus abundances for each strain can be found in Table S2.

### Burst Size

**Figure 7:**
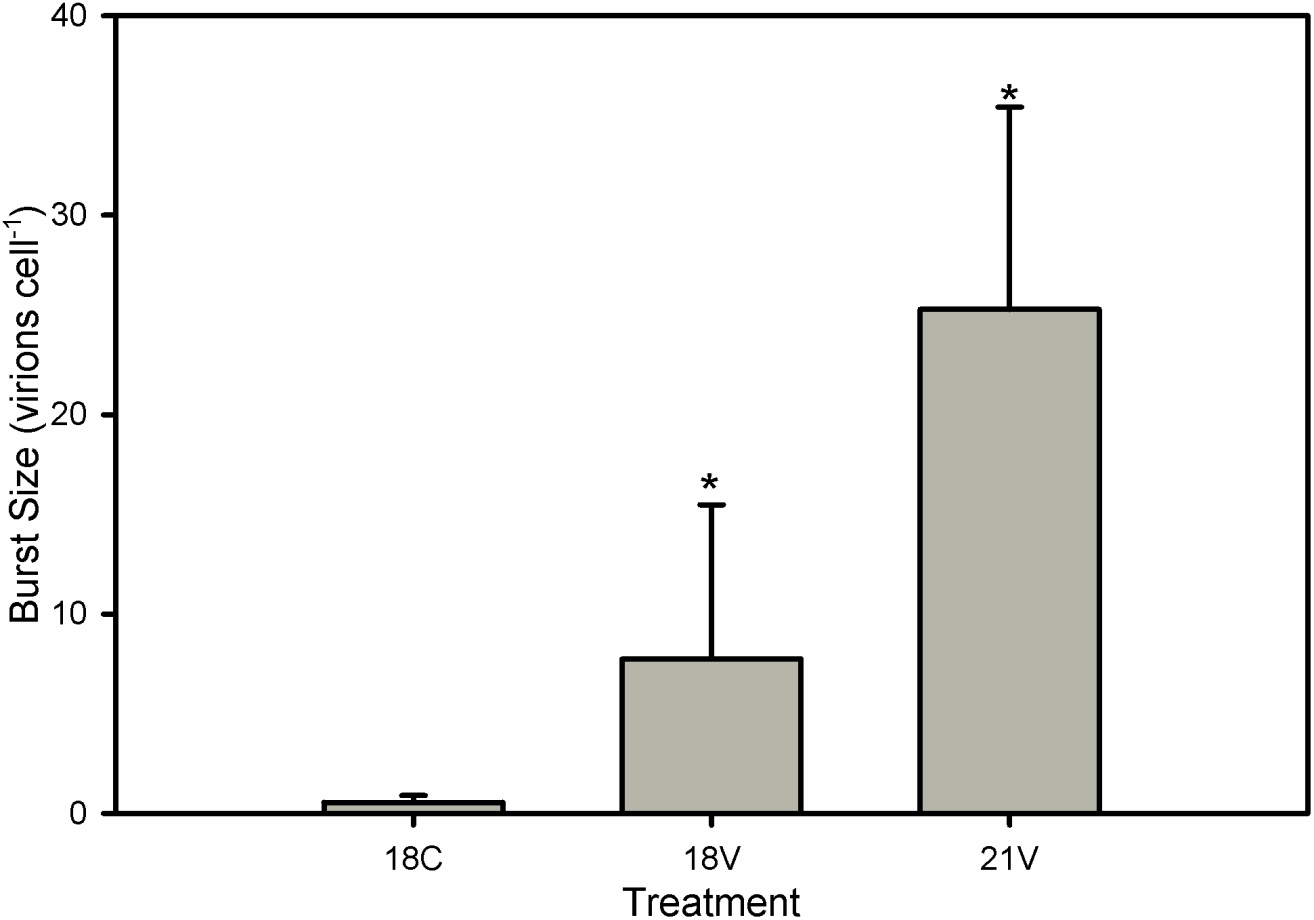
Viral Incubation. Burst Size (i.e. viral progeny produced per host cell lost calculated over a 24-hour period) for the viral incubation experiment on strain 374. Viral isolate was incubated overnight at 18°C and 21°C, then used to infect cells growing at 18°C in order to assess whether elevated temperature affected the host or the virus. “18V” and “21V” here represent viral incubation temperatures rather than host growth temperatures, and “18C” represents the uninfected control. Error bars represent one standard deviation, and Asterisks represent a significant difference from the control (p<0.05), but not between treatments compared via two-way ANOVA.

We also tested whether elevated temperature impacted the inherent infectivity of EhVs by incubating EhV86 virions overnight in the dark either at 18° or 21°C followed by infection of exponentially growing *E. huxleyi* 374 cells at a permissible infection temperature of 18°C. Both treatments had burst sizes that were significantly higher than the uninfected control following a 24 h incubation (Fig. 7), indicating that exposure of EhVs to elevated temperature alone was not the cause of the induced resistance, but rather tied with alterations of host properties.

**Figure 8:**
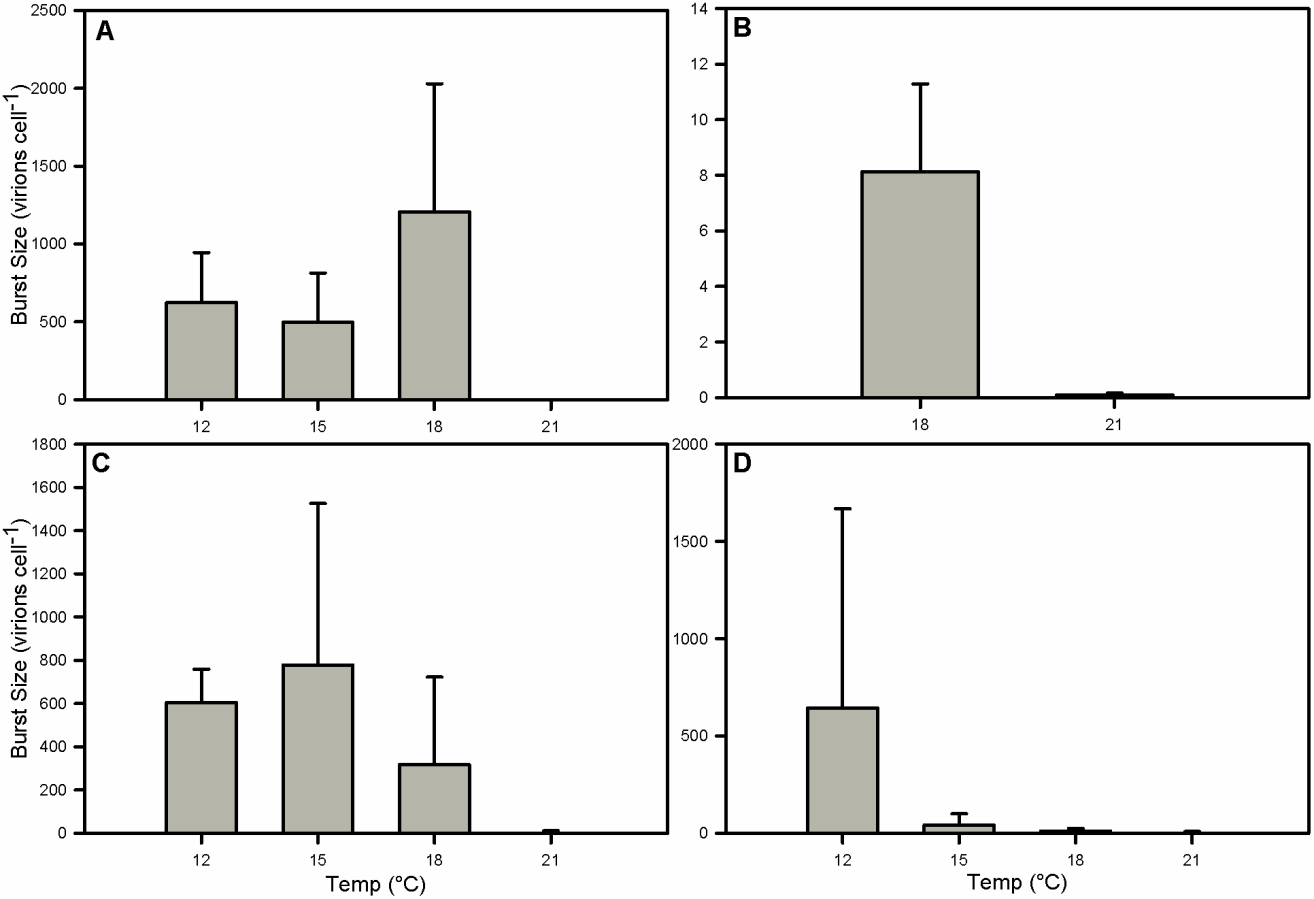
Burst Sizes. Burst Sizes for strains (A) CCMP 374, (B) CCMP 1516, (C) DWN 61/87/10, and (D) CCMP 392. Burst sizes were calculated using the 24-hour period between days 3 and 4 following infection except for CCMP1516 which were calculated from the 24-hour period between days 2 and 3, likely during the early infection chronic phase, explaining the atypically low values. Error bars represent one standard deviation. Within each strain no significant differences were found between infection-permissive temperatures. All comparisons made via two-way ANOVA.

Elevated temperature had a pronounced negative effect on lytic burst sizes in strains 374 (Fig. 8A), 1516 (Fig. 8B), 61/87/10 (Fig. 8C), and 392 (Fig. 8D). All strains tested were resistant to infection at 21°C and had a burst size of 0 virions cell^−1^ at that temperature. Strain 374 had very similar burst sizes at 12° and 15°C with means of 623 and 495 virions cell^−1^, respectively. In comparison, the burst size at 18°C was 1,206 virions cell^−1^, although not significantly different from 12°C and 15°C (Fig. 8A). Strain 1516 had a mean burst size of only 8 virions cell^−1^ in 18V (Fig. 8B), likely due to the calculations being performed on the 24-hour period between days 2 and 3, presumably during the chronic phase of infection, instead of days 3 and 4 as with other strains. Data from day 4 were not taken. Strain 61/87/10 exhibited a ∼50% lower burst size at 18°C than at 12°C or 15°C, although this result was not statistically significant (Fig. 8C). Burst sizes in 392, a strain thought to be virus-resistant, decreased, ranging from 644 virions per cell at 12°C to 42 at 15° and 11 at 18° (Fig. 8D). These burst sizes were not significantly different between temperature treatments; however, when considered in conjunction with virus and host abundance data the results are noteworthy. Data for the virus incubation experiment, and burst sizes for sensitive strains and the virus absorption experiments may be found in Table S3.

## Discussion

Given current climate change predictions of 1.5–3.0°C increases in SST by 2100 [35], it is critically important to understand how elevated temperatures impact *E. huxleyi*-EhV interactions and how they may manifest in the termination of *E. huxleyi* blooms. These impacts could have global significance for the biogeochemical cycling of carbon and sulfur. Using the IPCC temperature predictions and several *E. huxleyi*-EhV86 model systems as a platform, we tested the effect of a 3°C temperature increase on this globally-distributed and ecologically relevant host-virus system. Our results clearly demonstrate an elevated temperature-induced resistance to EhV infection in an array of sensitive host strains (CCMP374, CCMP1516, and DWN61/87/10). Though novel in *E. huxleyi*, similar temperature effects on infectivity/sensitivity have been demonstrated in the bloom-forming Rhaphidophyte, *Heterosigma akashiwo*-HaV system [17], and in terrestrial plant host/virus systems such as the Potato Leafroll Virus [44] and Soybean Mosaic Virus [45]. Together with the results from our study on *E. huxleyi*, it is clear that temperature plays a fundamental mechanistic role in host-virus interactions.

Notably, the temperature-induced resistant phenotype in *E. huxleyi* occurred without an increase in net DSS production. Observed increases in the dissolved pools of DMS, DMSP, and DMSO in 18V were most likely due to a release of particulate pools into the surrounding media upon virus-induced cell lysis. A comparison of dissolved DMS to dissolved DMSP, however, indicates that shifting of DMSP from the particulate to dissolved fraction outpaced conversion into DMS, possibly due to a bottleneck in DMSP lyase activity [29]. We interpret the decreases in the total pools of DMSP and DMSO to be partially caused by turnover due to scavenging of free oxygen radicals in an attempt to mitigate oxidative stress. Unfortunately, the lack of data for total DMS due to the presence of headspace in the experimental bottles precludes quantification of the rate of DMSP turnover in our axenic cultures. Hence, caution must be used in interpreting our DMS data as true values may be underestimated. Nonetheless, our DMS, DMSP_p_, and DMSP_d_ data corroborate those reported by Evans et al. [29], although they demonstrated an attenuated DMSP lyase activity during viral infection compared to uninfected controls. Our results are also consistent with the findings of Malin et al. [46], which determined that viral infection in *Phaeocystis pouchetii* increased DMS production. In conjunction with radical scavenging, this repressed DMSP-lyase activity may partially explain why dissolved DMSP concentration is an order of magnitude higher than dissolved DMS.

Viral termination of natural phytoplankton blooms represents an important mechanism by which the concentrations of DMSP_d_ can increase in seawater [7], [10], [11]. It has been speculated that bacterial degradation may be sufficient to completely convert a post-bloom DMSP_d_ pulse to DMS [11], suggesting that viral lysis and the release of intracellular DMSP may be even more climatologically significant than once thought, though other factors may also be important, such as DMS consumption rates [47]. Evans et al. [29] found that microzooplankton grazing of batch cultures leads to more DMS production than EhV-associated lysis. In the ocean, there are several additional mechanisms of DMSP cleavage including zooplankton grazing, algal senescence, bacterial degradation, and photo-oxidation. It is also important to note that, while viral lysis has been documented to cause *E. huxleyi* bloom termination [9], [12], [13], [16], it is difficult to say which may be the more important process for DMS release as grazers have been shown to preferentially feed on infected *E. huxleyi* cells [48]. In our experiments DMSP_d_ concentrations increased more than an order of magnitude relative to controls, a much greater increase than in DMS, though there was a marked increase in DMS production within the sensitive infected cultures. Given our results were derived from a simplified model system lacking microbial ecosystem complexity, they likely do not accurately reflect the total degree to which viral lysis may increase seawater DMS concentrations during bloom collapse. Nonetheless, the fact that DSS production does not directly increase with temperature corroborates the findings of van Rijssel and Gieskes [49], who questioned the positive climate feedback loop put forth in the CLAW hypothesis [5]. Our observed temperature-induced resistance phenotype cannot be explained by the ‘DMSP antiviral hypothesis’ [21], whereby DMSP cleavage into DMS and acrylic acid serves as a temporal antiviral mechanism, delaying viral mortality of *E. huxleyi* and bloom collapse and serving as a possible way to facilitate host-virus coexistence [20], [23], [28].

The hypothesis that temperature-induced differences in lipid content impart resistance to virus infection predicts changes in key lipid constituents and/or concentrations. Constitutive levels of sulfoquinovosyldiacylglycerol (SQDG), diacylglyceryltrimethylhomoserine (DGTS), and PG were consistently, though not significantly, lower at 21°C. DGTS levels did become significantly higher at 18°C by day 4. The lack of statistical significance may be due to natural variation that may be rectified by repeating the experiment with *E. huxleyi* 374 and other strains. Further tests should also include comparing lipid levels in resistant strains to those of susceptible strains as well as resistant and susceptible phenotypes within strains. A significant decrease in total IPLs occurred in 18V from a maximum of 6.9±0.4 fmol cell^−1^ on day 1 to a minimum of 4.0±0.3 fmol cell^−1^ on day 3 (Fig. 5), and was associated with a simultaneous loss in photosynthetic efficiency (Fig. 1C). For example, glycolipids such as digalactosyldiacylglycerol (DGDG) and monogalactosyldiacylglycerol (MGDG) are involved in chloroplast membrane function. In fact, MGDG as well as the sulfur-containing glycolipid, SQDG, appear to be restricted to thylakoid membranes in *E. huxleyi* [50], [51]. Coincident with the decrease in photosynthetic efficiency were significant decreases in MGDG and SQDG in 18V by day 2 and a smaller decrease in DGDG (Fig. 5). No large changes were observed in glycolipids in 18C, 21C and 21V experiments, consistent with our observation of high photosynthetic efficiency (Fig. 1C).

DGTS is a betaine lipid confined to plasma and chloroplast membranes [52]. Like PE and vGSL, DGTS increased significantly in 18V and no other treatment (Fig. 5). Diacylglyceryl carboxyhydroxymethylcholine (DGCC), an extraplastidial betaine lipid that may be involved in MGDG production [53], increased in 18V relative to other treatments becoming significantly higher by day 2 before falling below other treatments by day 4. These changes in betaine lipids are particularly interesting. The significant loss of DGCC in 18V relative to other treatments likely explains the decrease in MGDG. Incubation experiments of *Pavlova lutheri* with 14C-labelled oleic acid suggested a connection between the biosynthesis of DGCC and MGDG, leading to the proposal that DGCC was involved in the transfer of fatty acids to MGDG in the chloroplast [53]. The mechanism of transfer, however, remains unknown. Increase in DGTS in 18V could be correlated with changes in DMSP. For example, the synthesis pathways for both compounds involve methionine and N-methylation via S-adenosylmethionine [54], [55]. Increased DGTS could also be due to a simple replacement of PC lost to viral infection as observed in primitive vascular plants [54].

Given their mechanistic importance to different aspects of *E. huxleyi*-EhV interactions, [23], [27], [32], [33] we also specifically examined the levels of three GSL classes (hGSLs, vGSLs, and sGSLs). The healthy, host-derived hGSLs initially showed significant accumulation in successfully infected cultures, but dropped dramatically by day 4. Its production decreased with impaired photosynthetic productivity as evidenced by compromised F_v_/F_m_ on day 3. The decline in hGSL concentration in infected cells occurred concomitantly with an increase in vGSL accumulation, which indicates a retrenchment in GSL synthesis pathways toward virus-derived GSLs by day 3. Since vGSL synthesis is necessary for EhV production and host PCD [28], it is possible that initial increases in hGSL concentrations in infected cells represent a biochemical up-regulation of the host’s serine palmitoyl transferase, the first and rate-limiting enzyme in the ceramide biosynthesis pathway, as a competitive inhibition reaction with the EhV-derived serine palmitoyl transferase enzyme. This ostensible up-regulation is inconsistent with the notion that infected cells initially silence and/or implement transcriptional controls over host-derived sphingolipid synthesis genes [56].

A clear result from our study was the inhibitory effect that a 3°C increase in temperature had on EhV adsorption/absorption to host *E. huxleyi* cells, precluding successful infection. We confirmed that this temperature effect was not due to an alteration of the inherent EhV infectivity, as EhV86 virions exposed to overnight treatments of 18°C and 21°C retained their infectivity when incubated with host *E. huxleyi* cells at 18°C (Fig. 7). We were also able to eliminate the possibility that elevated temperature induced transition to a resistant haploid phase (i.e. ‘Cheshire Cat’ strategy [18]) by analyzing SYBR-stained flow cytograms. Changes in ploidy level would be represented by a decrease in both algal SYBR green fluorescence and side scatter profile. No such decreases were found in any treatment indicating no change in life stage.

Rather, the temperature-induced resistance and alteration of EhV absorption was at the level of host composition and/or physiology. EhVs appear to employ an entry and exit strategy through lipid rafts, chemically distinct membrane lipid microdomains that are enriched in GSLs and are involved in sensing extracellular stimuli and activating signaling cascades through protein-protein interactions [30], [32]. Lipidomic analyses of purified lipid rafts from control and EhV-infected *E. huxleyi* cells revealed two distinct host-derived GSL classes–sGSLs (sialic acid GSLs) and rGSLs (raft GSLs)–that were preferentially enriched in lipid rafts and closely corresponded to lipid raft physical and biochemical markers [32]. It is plausible that altered virus-sensitivity might be due to changes in these GSL classes.

Previous data revealed that sGSLs, which possess a sialic acid headgroup, are effective markers for susceptible *E. huxleyi* cells [33]. These lipids were relatively abundant in the four other sensitive strains tested and either absent or at trace levels in six resistant strains [33]. Possibly, sGSLs participate in viral attachment and/or release through the interaction with an EhV-encoded sialidase enzyme (ehv455) [31]. Here, the occurrence of sGSLs in all *E. huxleyi* 374 treatments, including the resistant 21V treatment, shows that infection dynamics depend upon additional non-IPL components that must be temperature sensitive.

Alternatively, the mechanism of resistance and reduced virus attachment might lie in fundamental changes to host cell surface receptors. For instance, proteomic analyses of purified lipid rafts from control and EhV-infected *E. huxleyi* cells revealed a variety of proteins affiliated with host recognition, defense, PCD, and innate immunity pathways [32]. These included: calmodulin-binding DENN/MADD domain-containing proteins that are involved in MAP kinase induction [57]; proline-rich extensins (PRICHEXTENSN), which function in the signal transduction of pathogen defense upon compromised cell wall structure [58], [59]; toll interleukin 1 receptor (TIR) and leucine-rich repeat (LRR) domain proteins, which are often connected by a nucleotide-binding (NB) domain and collectively mediate pathogen recognition/resistance and activate host-cell defense responses [60], [61]. TIR-NB-LRR proteins specifically recognize viral membrane proteins through ligand-receptor interaction resulting in the stress-induced, plant hypersensitive PCD response [62], [63]. An EhV86 C-type lectin 1 domain–containing membrane protein (ehv149; Q4A2Y5), a classic ligand-binding partner for toll-like receptors (TLR/TIR) reported in poxviruses and African swine fever virus (ASFV) [64], [65], was also detected in purified lipid rafts, implicating a possible protein-protein (TIR-NB-LRR/C-type Lectin) specific binding interaction for the successful attachment, entry, and exit through lipid rafts. Given this mechanistic role, we hypothesize that elevated temperature repressed the expression of TIR-NB-LRR proteins, but further verification is warranted using relevant information from the *E. huxleyi* genome [31], [32], [66].

While we were not able to definitively identify the precise mechanism of temperature-induced EhV resistance in *E. huxleyi,* our study highlights the physiological plasticity of this host-virus relationship within a realistic IPCC-projected global temperature range over the next century (+3°C). This area of research is of particular importance to understand and model current and future changes in the earth’s climate. Given the global prevalence of EhV-termination of *E. huxleyi* blooms and that all sensitive cells displayed a general resistance to EhV infection when incubated at these elevated temperatures (and also that the resistant strain 392 displayed potential EhV production at lower temperatures), it is tempting to speculate how this plasticity will play out in natural populations. It will likely be determined by the relative balance between the rate of sea surface temperature rise and the tempo of the co-evolutionary arms race between *E. huxleyi*-EhV [27]. On balance, the latter would serve as a mechanism to adapt to host-derived changes and possibly mute the ecological impact of widespread resistance. Given the observed release of DSS species (this study) and greatly enhanced production of transparent exopolymeric particles (TEP) [23] upon EhV infection, which can greatly facilitate carbon export to deep waters, this may have profound biogeochemical implications for S and C cycles in the upper ocean such as disrupted transport of biogenic sulfur to the atmosphere and sequestration of carbon.

## Conclusion

Our results demonstrate that a 3°C increase in temperature induced a profound shift towards viral resistance in an array of normally sensitive *E. huxleyi* strains. Significant changes in DSS pool sizes occurred during infection at 18°C, but in neither treatment at 21°C indicating that the antioxidant capacity of DSS is likely not the mechanism responsible for the resistant phenotype. We did, however, confirm that elevated DMS production is a symptom of successful viral infection [46]. This does not necessarily contradict the DMSP antiviral hypothesis, which posits that DSS from infected cells may reduce infectivity of viral progeny and delay bloom termination [21]. Successful viral infection imparted major changes in the polar lipid content of host cells consistent with previous observations [32], [33]. Our work implicates fundamental cellular changes in the host at the level of adsorption of EhVs to the cell surface as the mechanism behind temperature induced resistance in *E. huxleyi*. Future work should investigate the role of temperature in imparting viral resistance through changes in lipid rafts and their associated receptor and host response proteins.

## Supporting Information

Figure S1 **Transmission Electron Microscopy.** TEM images of E. huxleyi cells from control treatments 18C (A) and 21C (B) taken on day 2 of sampling.
(TIF)

Figure S2 **Total DMSO.** Total DMSO concentrations showing a 30% decrease in 18V between days 2 and 3, all DMSO_t_ concentrations showed a net 3.8-fold increase over the course of the experiment. Error bars represent one standard deviation, and asterisks represent statistical significance according to a Holm-Sidak t-test (p<0.05).
(TIF)

Figure S3 **Dynamics of Cell and Virus Abundance for Resistant Strains.** Cell and viral abundance for E. huxleyi strain (panels A and B, respectively) CCMP 373, (C and D, respectively) CCMP 379, and (E and F, respectively) CCMP 392 at 18° and 21°C. There was no significant loss of cell abundance or accumulation of viral particles indicating all three strains were virus resistant at both temperatures. Error bars represent one standard deviation.
(TIF)

Table S1 **Cell Abundances, Virus Abundances, Host Health, Dimethylated Sulfur Species, and Intact Polar Lipids.** Data values for Figures 1, 3, 5, and S1. All lipid data are in fmol cell^−1^.
(XLS)

Table S2 **Dynamics of Cell and Virus Abundance for Additional Strains.** Data values for Figures 6 and S2.
(XLS)

Table S3 **Virus Incubation and Burst Sizes.** Data values for Figures 4, 7, and 8.
(XLS)

## References

[1] Berge G (1962) Discolouration of the sea due to *Coccolithus huxleyi* “bloom”. Sarsia 6: 27–40.

[2] Tyrell T, Merico A (2004) *Emiliania huxleyi*, bloom observations and the conditions that induce them. In: Thierstein HR, Young JR editors. Coccolithophores: From Molecular Processes to Global Impact. Springer Berlin Heidelberg, New York. 75–97.

[3] Todd JD, Rogers R, Li YG, Wexler M, Bond PL, et al (2007) Structural and regulatory genes required to make the gas dimethyl sulfide in bacteria. Science 315: 666–669. 17272727 10.1126/science.1135370

[4] Todd JD, Curson ARJ, Kirkwood M, Sullivan MJ, Green RT, et al (2011) DddQ, a novel, cupin-containing, dimethylsulfoniopropionate lyase in marine roseobacters and in uncultured marine bacteria. Environ Microbiol 13(2): 427–438. 20880330 10.1111/j.1462-2920.2010.02348.x

[5] Charlson RJ, Lovelock JE, Andreae MO, Warren SG (1987) Oceanic phytoplankton, atmospheric sulphur, cloud albedo and climate. Nature 326(16): 655–661.

[6] Ayers GP, Ivey JP, Gillett RW (1991) Coherence between seasonal cycles of dimethyl sulphide, methanesulphonate and sulphate in marine air. Nature 349: 404–406.

[7] Bratbak G, Levasseur M, Michaud S, Cantin G, Fernandez E, et al (1995) Viral activity in relation to *Emiliania huxleyi* blooms: a mechanism of DMSP release? Mar Ecol Prog Ser 128: 133–142.

[8] Schroeder DC, Oke J, Malin G, Wilson WH (2002) Coccolithovirus (*Phycodnaviridae*): Characterisation of a new large dsDNA algal virus that infects *Emiliania huxleyi* . Arch Virol 147: 1685–1698. 12209309 10.1007/s00705-002-0841-3

[9] Bratbak G, Wilson W, Heldal M (1996) Viral control of *Emiliania huxleyi* blooms? J Mar Sys 9: 75–81.

[10] Fuhrman JA (1999) Marine viruses and their biogeochemical and ecological effects. Nature 399: 541–548. 10376593 10.1038/21119

[11] Hill RW, White BA, Cottrell MT, Dacey JWH (1998) Virus-mediated total release of dimethylsulfoniopropionate from marine phytoplankton: a potential climate process. Aquat Microb Ecol 14: 1–6.

[12] Jacquet S, Heldal M, Iglesias-Rodriquez D, Larsen A, Wilson W, et al (2002) Flow cytometric analysis of an *Emiliania huxleyi* bloom terminated by viral infection. Aquat Microb Ecol 27: 111–24.

[13] Wilson WH, Tarran G, Zubkov MV (2002) Viral Dynamics in a coccolithophore-dominated bloom in the North Sea. Deep-Sea Res pt II 49: 2951–2963.

[14] Suttle CA (2005) Viruses in the sea. Nature 437: 356–61. 16163346 10.1038/nature04160

[15] Suttle CA (2007) Marine viruses–major players in the global ecosystem. Nature Rev Microbiol 5(10): 801–812. 17853907 10.1038/nrmicro1750

[16] Lehahn Y, Koren I, Schatz D, Frada F, Sheyn U, et al (2014) Decoupling physical from biological processes to assess the impact of viruses on a mesoscale algal bloom. Curr Biol 24: 2041–2046. 25155511 10.1016/j.cub.2014.07.046

[17] Nagasaki K, Yamaguchi M (1998) Effect of temperature on the algicidal activity and the stability of HaV (*Heterosigma akashiwo* Virus). Aquat Microb Ecol 15: 211–216.

[18] Frada M, Probert I, Allen MJ, Wilson WH, de Vargas C (2008) The “Cheshire Cat” escape strategy of the coccolithophore *Emiliania huxleyi* in response to viral infection. Proc Nat Acad Sci 105(41): 15944–15949. 18824682 10.1073/pnas.0807707105 PMC2572935

[19] Frada MJ, Bidle KD, Probert I, Vargas CD (2012) In situ survey of life cycle phases of the coccolithophore *Emiliania huxleyi* (Haptophyta). Environ Microbiol 14: 1558–1569. 22507290 10.1111/j.1462-2920.2012.02745.x

[20] Bidle KD, Haramaty L, Barcelos e Ramos J, Falkowski P (2007) Viral activation and recruitment of metacaspases in the unicellular coccolithophore, *Emiliania huxleyi* . Proc Nat Acad Sci 104: 6049–6054. 17392426 10.1073/pnas.0701240104 PMC1838821

[21] Evans C, Malin G, Wilson WH, Liss PS (2006) Infectious titers of *Emiliania huxleyi* Virus 86 are reduced by exposure to millimolar dimethyl sulfide and acrylic acid. Limnol Oceanogr 51(5): 2468–2471.

[22] Evans C, Malin G, Mills GP, Wilson WH (2006) Viral infection of *Emiliania huxleyi* (Prymnesiophyceae) leads to elevated production of reactive oxygen species. J Phycol 42: 1040–1047.

[23] Vardi A, Haramaty L, Van Mooy BAS, Fredricks HF, Kimmance SA, et al (2012) Host-virus dynamics and subcellular controls of cell fate in a natural coccolithophore population. Proc Nat Acad Sci 109(47): 19327–19332. 23134731 10.1073/pnas.1208895109 PMC3511156

[24] Sunda W, Kieber DJ, Kiene RP, Huntsman S (2002) An antioxidant function for DMSP and DMS in marine algae. Nature 418: 317–320. 12124622 10.1038/nature00851

[25] Steinke M, Wolfe GV, Kirst GO (1998) Partial characterisation of dimethylsulphoniopropionate (DMSP) lyase isozymes in 6 strains of *Emiliania huxleyi.* . Mar Ecol Prog Ser 175: 215–225.

[26] Spiese CE, Kieber DJ, Nomura CT, Kiene RP (2009) Reduction of dimethylsulfoxide by marine phytoplankton. Limnol Oceanogr 54(2): 560–570.

[27] Bidle KD, Vardi A (2011) A chemical arms race at sea mediates algal host-virus interactions. Curr Opin Microbiol 14: 449–457. 21816665 10.1016/j.mib.2011.07.013

[28] Vardi A, Van Mooy BAS, Fredricks HF, Popendorf KJ, Ossolinski JE, et al (2009) Viral Glycosphingolipids induce lytic infection and cell death in marine phytoplankton. Science 326: 861–865. 19892986 10.1126/science.1177322

[29] Evans C, Kadner SV, Darroch LJ, Wilson WH, Liss PS, et al (2007) The relative significance of viral lysis and microzooplankton grazing as pathways of dimethylsulfoniopropionate (DMSP) cleavage: an *Emiliania huxleyi* culture study. Limnol Oceanogr 52(3): 1036–1045.

[30] Mackinder LCM, Worthy CA, Biggi G, Hall M, Ryan KP, et al (2009) A unicellular algal virus, *Emiliania huxleyi* Virus 86, exploits an animal-like infection strategy. J Gen Virol 90: 2306–2316. 19474246 10.1099/vir.0.011635-0

[31] Wilson HW, Schroeder DC, Allen MJ, Holden MTG, Parkhill J, et al (2005) Complete genome sequence and lytic phase transcription profile of a *Coccolithovirus* . Science 309: 1090–1092. 16099989 10.1126/science.1113109

[32] Rose SL, Fulton JM, Brown CM, Natale F, Van Mooy BAS, Bidle KD (2014) Isolation and characterization of lipid rafts in *Emiliania huxleyi*: a role for membrane microdomains in host-virus interactions. Environ Microbiol 16: 1150–1166. 24330022 10.1111/1462-2920.12357

[33] Fulton JM, Fredricks HF, Bidle KD, Vardi A, Kendrick BJ, et al (2014) Novel molecular determinants of viral susceptibility and resistance in the lipidome of *Emiliania huxleyi* . Environ Microbiol 16: 1137–1149. 24330049 10.1111/1462-2920.12358

[34] Suzuki T, Suzuki Y (2006) Lipid dynamics and pathobiology in membrane lipid rafts. Biol Pharm Bull 29(8): 1538–1541. 16880600 10.1248/bpb.29.1538

[35] Intergovernmental Panel on Climate Change (2013) Climate Change 2013: The Physical Science Basis. Contribution of Working Group I to the Fifth Assessment Report of the Intergovernmental Panel on Climate Change. Stocker TF, Qin D, Plattner G-K, Tignor M, Allen SK, et al., editors Cambridge, United Kingdom and New York, NY, USA: Cambridge University Press.

[36] Hochachka PW, Somero GN (2002) Biochemical adaptation: mechanism and process in physiological evolution. Oxford University Press, New York, NY.

[37] Brussaard CPD, Payet JP, Winter C, Weinbauer MG (2010) Quantification of aquatic viruses by flow cytometry. In: Wilhelm SW, Weinbauer MG, Suttle CU, editors. Manual of Aquatic Viral Ecology: American Society of Limnology and Oceanography. 102–9.

[38] DiTullio GR, Smith Jr WO (1995) Relationship between dimethylsulfide and phytoplankton pigment concentrations in the Ross Sea, Antarctica. Deep Sea Res Pt I 42(6): 873–92.

[39] Riseman SF, DiTullio GR (2004) Particulate dimethylsulfoniopropionate and dimethylsulfoxide in relation to iron availability and algal community composition structure in the Peru Upwelling System. Can J Fish Aquat Sci 61: 721–35.

[40] Van Mooy BAS, Fredricks HF (2010) Bacterial and eukaryotic intact polar lipids in the eastern subtropical South Pacific: water-column distribution, planktonic sources, and fatty acid composition. Geochim Cosmochim Acta 74: 6499–6516.

[41] Bligh EG, Dyer WJ (1959) A rapid method for total lipid extraction and purification. Can J Biochem Physiol 37: 911–917. 13671378 10.1139/o59-099

[42] Popendorf KJ, Fredricks HF, Van Mooy BAS (2013) Molecular ion-independent quantification of polar glycerolipid classes in marine plankton using tripe quadrupole MS. Lipids 48(2): 185–195. 23269556 10.1007/s11745-012-3748-0

[43] Keller MD, Bellows WK, Guillard RRL (1989) Dimethyl sulfide production in marine phytoplankton. In: Saltzman ES, Cooper WJ, editors. Biogenic Sulfur in the Environment: American Chemical Society. 167–182.

[44] Syller J (1991) The effects of temperature on the susceptibility of potato plants to infection and accumulation of potato leafroll virus. J Phytopath 133: 216–224.

[45] Mansky LM, Durand DP, Hill JH (1991) Effects of temperature on the maintenance of resistance to soybean mosaic virus in soybean. Phytopathology 81: 535–538.

[46] Malin G, Wilson WH, Bratbak G, Liss PS, Mann NH (1998) Elevated production of dimethylsulfide resulting from viral infection of cultures of *Phaeocystis pouchetii.* . Limnol Oceanogr 43: 1289–1293.

[47] Kiene RP, Bates TS (1990) Biological removal of dimethyl sulphide from sea water. Nature 345: 702–705.

[48] Evans C, Wilson WH (2008) Preferential grazing of *Oxyrrhis marina* on virus-infected *Emiliania huxleyi* Limnol Oceanogr. 53: 2035–2040.

[49] van Rijssel M, Gieskes WWC (2002) Temperature, light, and the dimethylsulfoniopropionate (DMSP) content of *Emiliania huxleyi* (Prymnesiophyceae). J Sea Res 48: 17–27.

[50] Goss R, Wilhelm C (2009) Lipids in algae, lichens, and mosses. In: Wada H, Murata N, editors. Lipids in Photosynthesis: Essential and Regulatory Functions: Springer Science + Business Media B. V: 117–135.

[51] Bell MV, Pond D (1996) Lipid composition during growth of motile and coccolith forms of *Emiliania huxleyi.* . Phytochemistry 41(2): 465–471.

[52] Sato N (1992) Betaine Lipids. Bot Mag Tokyo 105: 185–197.

[53] Eichenberger W, Gribi C (1997) Lipids of *Pavlova lutheri*: cellular site and metabolic role of DGCC. Phytochemistry 45(8): 1561–1567.

[54] Riekhof WR, Sears BB, Benning C (2005) Two enzymes, BtaA and BtaB, are sufficient for betaine lipid biosynthesis in bacteria. Arch Biochem Biophys 441: 96–105. 16095555 10.1016/j.abb.2005.07.001

[55] Stefels J (2000) Physiological aspects of the production and conversion of DMSP in marine algae and higher plants. J Sea Res 43: 183–197.

[56] Pagarete A, Allen MJ, Wilson WH, Kimmance SA, de Vargas C (2009) Host-Virus shift of the sphingolipid pathway along an *Emiliania huxleyi* bloom: survival of the fattest. Environ microbial 11(11): 2840–2848. 10.1111/j.1462-2920.2009.02006.x 19638172

[57] Schievella AR, Chen JH, Graham JR, Lin L-L (1997) MADD, a Novel Death Domain Protein That Interacts with the Type I Tumor Necrosis Factor Receptor and Activates Mitogen-activated Protein Kinase. J Biol Chem 272: 12069–12075. 9115275 10.1074/jbc.272.18.12069

[58] Sanabria NM, Huang J-C, Dubery IA (2010) Self/nonself perception in plants in innate immunity and defense. Self Nonself 1: 40–54. 21559176 10.4161/self.1.1.10442 PMC3091600

[59] Silva NF, Goring DR (2002) The proline-rich, extensin-like receptor kinase-1 (PERK1) gene is rapidly induced by wounding. Plant Mol Biol 50: 667–85. 12374299 10.1023/a:1019951120788

[60] Peart JR, Mestre P, Lu R, Malcuit I, Baulcombe DC (2005) NRG1, a CC-NB-LRR protein, together with N, a TIR-NB-LRR, mediates resistance against Tobacco Mosaic Virus. Curr Biol 15: 968–73. 15916955 10.1016/j.cub.2005.04.053

[61] Swiderski MR, Birker D, Jones JDG (2009) The TIR Domain of TIR-NB-LRR resistance proteins is a signaling domain involved in cell death induction. Mol Plant Microbe Interact 22: 157–65. 19132868 10.1094/MPMI-22-2-0157

[62] Heath MC (2000) Hypersensitive response-related death. Plant Mol Biol 44: 321–34. 11199391 10.1023/a:1026592509060

[63] Nimchuk Z, Eulgem T, Holt III BF, Dangl JL (2003) Recognition and response in the plant immune system. Ann Rev Genet 37: 579–609. 14616074 10.1146/annurev.genet.37.110801.142628

[64] Neilan JG, Borca MV, Lu G, Kutish GF, Kleiboeker SB, et al (1999) An African swine fever virus ORF with similarity to C-type lectins is non-essential for growth in swine macrophages in vitro and for virus virulence in domestic swine. J Gen Virol 80: 2693–97. 10573162 10.1099/0022-1317-80-10-2693

[65] Cambi A, Koopman M, Figdor CG (2005) How C-type lectins detect pathogens. Cell Microbiol 7: 481–88. 15760448 10.1111/j.1462-5822.2005.00506.x

[66] Kegel JU, John U, Valentin K, Frickenhaus S (2013) Genome variations associated with viral susceptibility and calcification in *Emiliania huxleyi* . PLoS One 8(11): e80684 doi:10.1371/journal.pone.0080684 24260453 10.1371/journal.pone.0080684 PMC3834299

