# Supplementary figures and images for: Temperature-Induced Viral Resistance in Emiliania huxleyi (Prymnesiophyceae)
Source: PLoS One. 2014 Nov 18;9(11):e112134. doi: 10.1371/journal.pone.0112134 (PMC4236053; doi:10.1371/journal.pone.0112134)

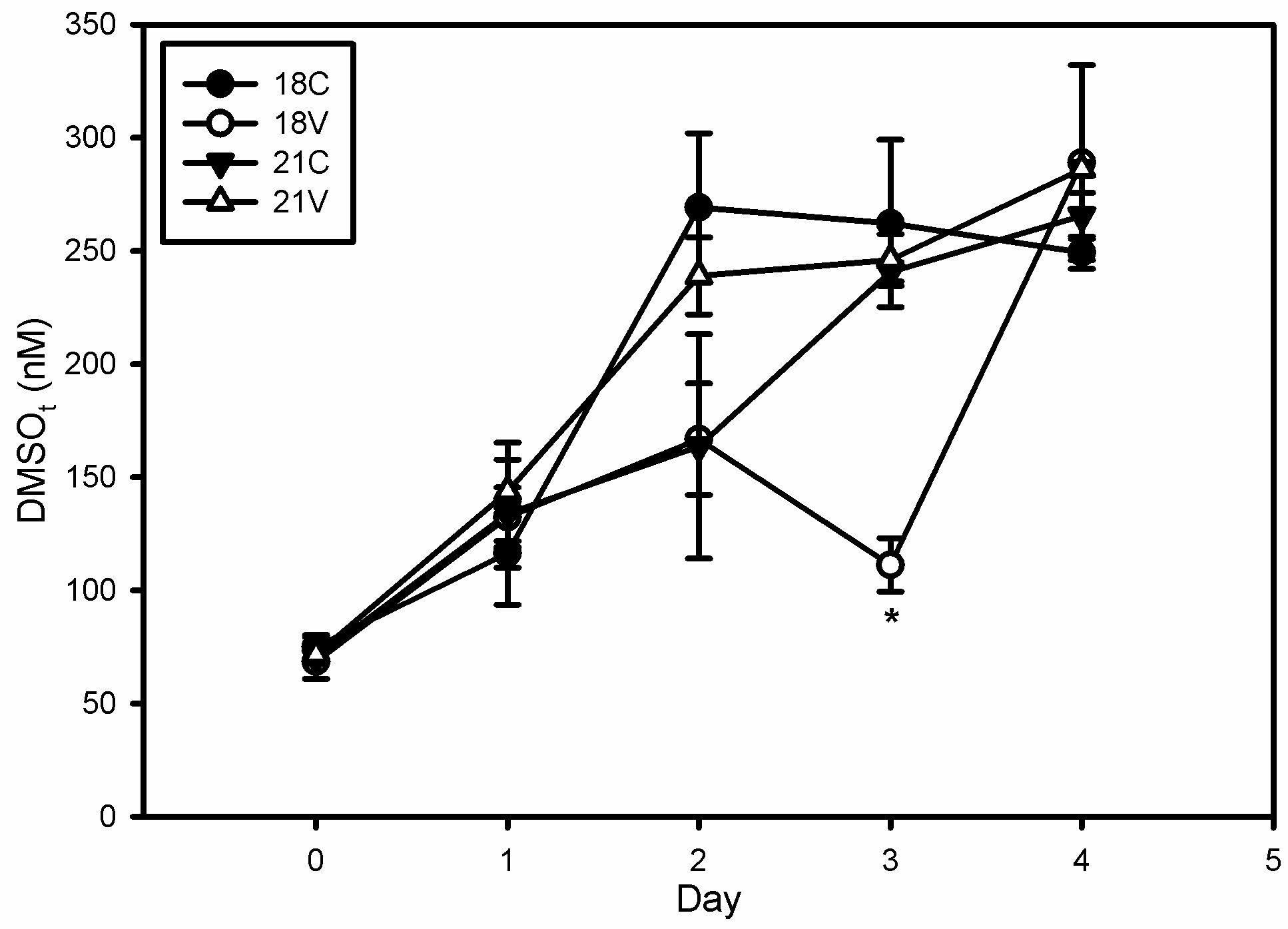

Supplement: Figure S1 — Transmission Electron Microscopy. TEM images of E. huxleyi cells from control treatments 18C (A) and 21C (B) taken on day 2 of sampling. (TIF) [file pone.0112134.s001.tif]

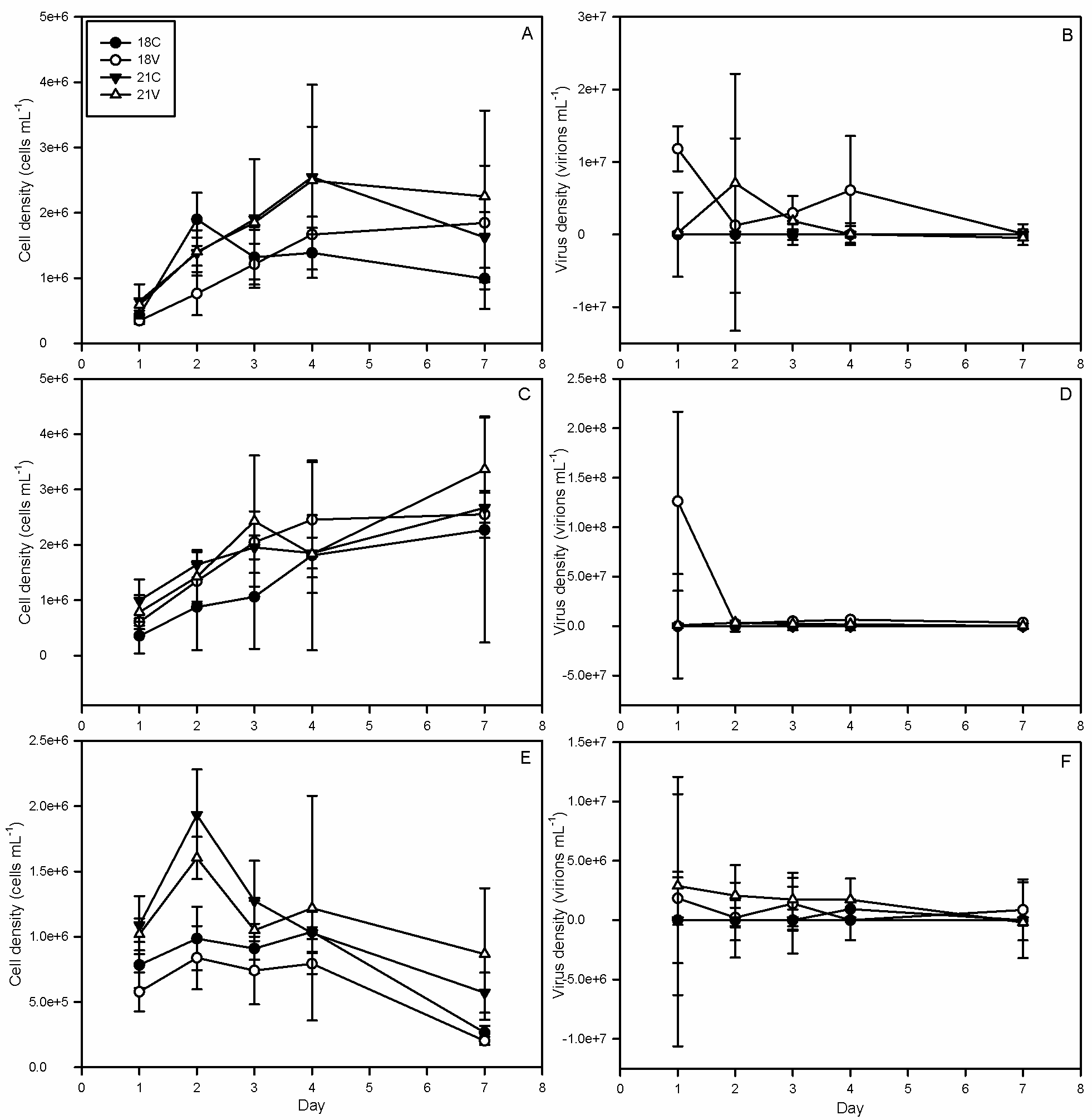

Supplement: Figure S2 — Total DMSO. Total DMSO concentrations showing a 30% decrease in 18V between days 2 and 3, all DMSOt concentrations showed a net 3.8-fold increase over the course of the experiment. Error bars represent one standard deviation, and asterisks represent statistical significance according to a Holm-Sidak t-test (p<0.05). (TIF) [file pone.0112134.s002.tif]

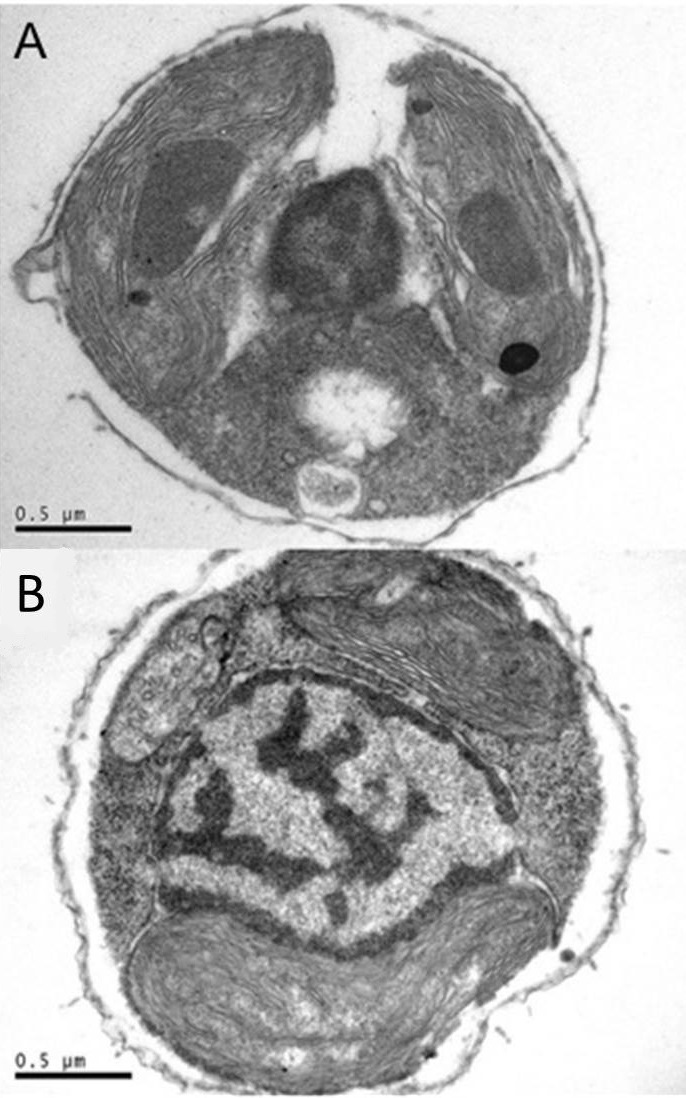

Supplement: Figure S3 — Dynamics of Cell and Virus Abundance for Resistant Strains. Cell and viral abundance for E. huxleyi strain (panels A and B, respectively) CCMP 373, (C and D, respectively) CCMP 379, and (E and F, respectively) CCMP 392 at 18° and 21°C. There was no significant loss of cell abundance or accumulation of viral particles indicating all three strains were virus resistant at both temperatures. Error bars represent one standard deviation. (TIF) [file pone.0112134.s003.tif]
